# Characterization of Antigen-Induced CD4+ T-Cell Senescence in Multiple Sclerosis

**DOI:** 10.3389/fneur.2022.790884

**Published:** 2022-02-03

**Authors:** Paula Tomas-Ojer, Marco Puthenparampil, Carolina Cruciani, María José Docampo, Roland Martin, Mireia Sospedra

**Affiliations:** ^1^Neuroimmunology and MS Research (NIMS), Department of Neurology, University Hospital and University Zurich, Zurich, Switzerland; ^2^Department of Neuroscience DNS, University-Hospital of Padova, Padova, Italy

**Keywords:** multiple sclerosis (MS), CD4, T-cell, T-cell senescence, regulation

## Abstract

Antigen-induced T-cell exhaustion and T-cell senescence are peripheral regulatory mechanisms that control effector T-cell responses. Markers of exhaustion and senescence on T Cells indicate the previous activation by repetitive stimulation with specific antigens. Malignant tumors are accompanied by enhanced T-cell exhaustion and T-cell senescence resulting in immune evasion, while these control mechanisms might be diminished in autoimmune diseases including multiple sclerosis (MS). To better understand the involvement of antigen-induced T-cell senescence in controlling CD4+ T-cell-mediated autoimmune responses in MS, we have analyzed the re-expression of CD45RA and the downregulation of CD28 and CD27 molecules as markers of antigen-induced T-cell senescence in fresh cerebrospinal fluid (CSF)-infiltrating and paired circulating T cells from patients with MS. Patients with different levels of CD4+ T-cell senescence were identified and characterized regarding demographical and clinical features as well as intrathecal markers of neurodegeneration. CD4+ T-cell senescence was also analyzed in control patients to explore a putative deficit of this regulatory mechanism in MS. This study shows heterogeneity of markers of CD4+ T-cell senescence in patients with MS. Patients with high levels of CD4+ T-cell senescence in peripheral blood showed increased frequencies of CSF-infiltrating CD28+ CD27-EM CD4+ T cells with a proinflammatory Th1 functional phenotype. The correlation of these cells with the intrathecal levels of neurofilament light chain, a marker of neurodegeneration, suggests their relevance in disease pathogenesis and the involvement of T-cell senescence in their regulation. Markers of antigen-induced T-senescence, therefore, show promise as a tool to identify pathogenic CD4+ T cells in patients with MS.

## Introduction

T cells are crucial elements of the adaptive immune system to protect us from pathogens and tumors. The potent effector functions of T cells assure protection, but also can represent a risk for self-tissues, if they are not tightly regulated (1). The mechanisms involved in T-cell control include a negative selection in the thymus that prevents the differentiation of T cells with strong reactivity against autoantigens and several peripheral mechanisms that restrain the magnitude and timing of T-cell responses. These regulatory mechanisms can be T-cell intrinsic because they act directly on the responding T cells and T-cell extrinsic because they depend on other cell subsets, such as regulatory T cells. The T-cell-intrinsic regulatory mechanisms, or checkpoints, control all the T-cell differentiation stages. In naïve T cells, tolerance is maintained by quiescence and ignorance as well as by anergy induced by deficient costimulation during T-cell activation. In effector T cells, the main peripheral tolerance checkpoints are T-cell exhaustion and T-cell senescence induced by repetitive antigen stimulation. Antigen-induced exhausted T cells display reduced responses to antigens and are characterized by decreased cytokine production and high expression of inhibitory receptors, such as programmed cell death protein 1 (PD-1). T-cell senescence is a cell stage, in which cells do not divide anymore as a consequence of telomere shortening and/or DNA damage. Antigen-induced senescent T cells are characterized by telomere erosion, re-expression of CD45RA, downregulation of CD28 and CD27 expression, and increased production of proinflammatory cytokines (2). Other factors, such as oxygen species or ionizing radiation can also induce T-cell senescence by damaging DNA, but the role of this telomer-independent T-cell senescence in maintaining peripheral T-cell tolerance is unknown (1).

An excess of antigen-induced T-cell exhaustion and senescence has been associated with chronic infections (3) and the development of tumors (4). Malignant tumors often promote exhaustion of tumor-infiltrating T cells (TILs) *via* the PD-1/PDL-1 pathway, and PD-1 or PDL-1 target therapies have beneficial effects on several tumors. Tumors also induce markers of T-cell senescence in TILs, such as downregulation of CD27 and CD28 costimulatory molecules. These molecules are required for efficient immune response and are necessary for effective PD1-directed therapy (5, 6). Interestingly, PD-1/PDL-1 targeting therapies have been associated with the adverse development of acute autoimmune reactions and the onset of autoimmune diseases (7). In contrast to cancer or chronic infections, autoimmunity might be associated with reduced T-cell exhaustion since the presence of exhausted T cells has been linked to more favorable clinical outcomes in different autoimmune diseases including MS (8–12). MS is an autoimmune disease of the central nervous system (CNS) (13), in which the expression of PDL-1 in brain lesions (14), the association of PD-1 gene polymorphisms with disease progression (15), the downregulation of PD-1/PD-L1 on peripheral blood mononuclear cells (16), and the increased frequency of PD1+ T cells in patients during remission (12) support an involvement of T-cell exhaustion in disease pathogenesis. Regarding T-cell senescence, premature or accelerated aging that includes immune senescence of different cell types has been described in several autoimmune diseases including MS (17–19). However, the involvement of antigen-induced T-cell senescence in MS and other autoimmune diseases remains unclear.

B cells (20), CD8+ T cells (21, 22), and, particularly, autoreactive CD4+ T cells (13) play a central role in pathogenesis of MS. To better understand the involvement of antigen-induced T-cell senescence in controlling CD4+ T-cell-mediated autoimmune responses in MS, we have analyzed the re-expression of CD45RA and the downregulation of CD28 and CD27 as markers of antigen-induced T-cell senescence (23–25) in fresh cerebrospinal fluid (CSF)-infiltrating and paired circulating T cells from patients with MS. Based on this analysis, patients with different levels of CD4+ T-cell senescence were identified and characterized regarding demographical and clinical features and also intrathecal markers of neurodegeneration. A putative weakness in this regulatory mechanism in MS has also been addressed by comparing intrathecal and peripheral CD4+ T-cell senescence in patients with MS and controls affected of other inflammatory and non-inflammatory neurological diseases.

## Materials and Methods

### Patient Material

Cerebrospinal fluid and paired blood samples obtained for diagnostic purposes were collected from 50 untreated patients with MS, 12 control patients affected by other non-inflammatory neurological diseases (ONINDs), and 12 control patients affected by other inflammatory neurological diseases (OINDs). Patient characteristics are shown in Supplementary Table S1. All the patients were recruited at the Neuroimmunology and MS Research Section, Neurology Clinic, University Hospital Zurich (USZ). Diagnosis of MS was based on the revised McDonald criteria (26). The study procedures were approved by the Cantonal Ethics Committee of Zurich (EC-No. 2013-0001) and all the patients or relatives signed informed consent.

### Flow Cytometric Immunophenotyping

Cerebrospinal fluid infiltrating and paired circulating cells were immunophenotyped using flow cytometry as previously reported (27). In brief, CSF-infiltrating cells (>10,000 cells in the first hour after collection) and blood circulating cells obtained from 800 ml of peripheral blood after lysis of red blood cells (RBCs) using RBC lysis buffer (BioLegend, San Diego, California, USA) were stained with a cocktail of 13 monoclonal antibodies. Samples were acquired in an LSR Fortessa cytometer (BD Biosciences, Franklin Lakes, New Jersey, USA) and analyzed using FACSDiva (BD) and FlowJO (TreeStar Incorporation, Ashland, Oregon, USA) software. Gatting strategy is shown in Supplementary Figure S1.

### Enzyme-Linked Immunosorbent Assays

The amount of neurofilament light chain (NF-L) and chitinase 3-like 1 (CHI3L1) proteins were quantified in CSF samples using ELISA (Human Diagnostics, Umea, Sweden and MicroVue, Athens, Ohio, USA, respectively) according to the instructions of the manufacturer.

### Statistics

To compare more than two variables, we used the Kruskal–Wallis test for non-normally distributed variables. Linear correlation between variables was tested using the Spearman rank correlation coefficient for non-normally distributed variables. The significance level was set at *p* < 0.05.

## Results

### Characterization of CD4+ T-Cell Senescence Based on the Re-expression of CD45RA

**Figure 1:**
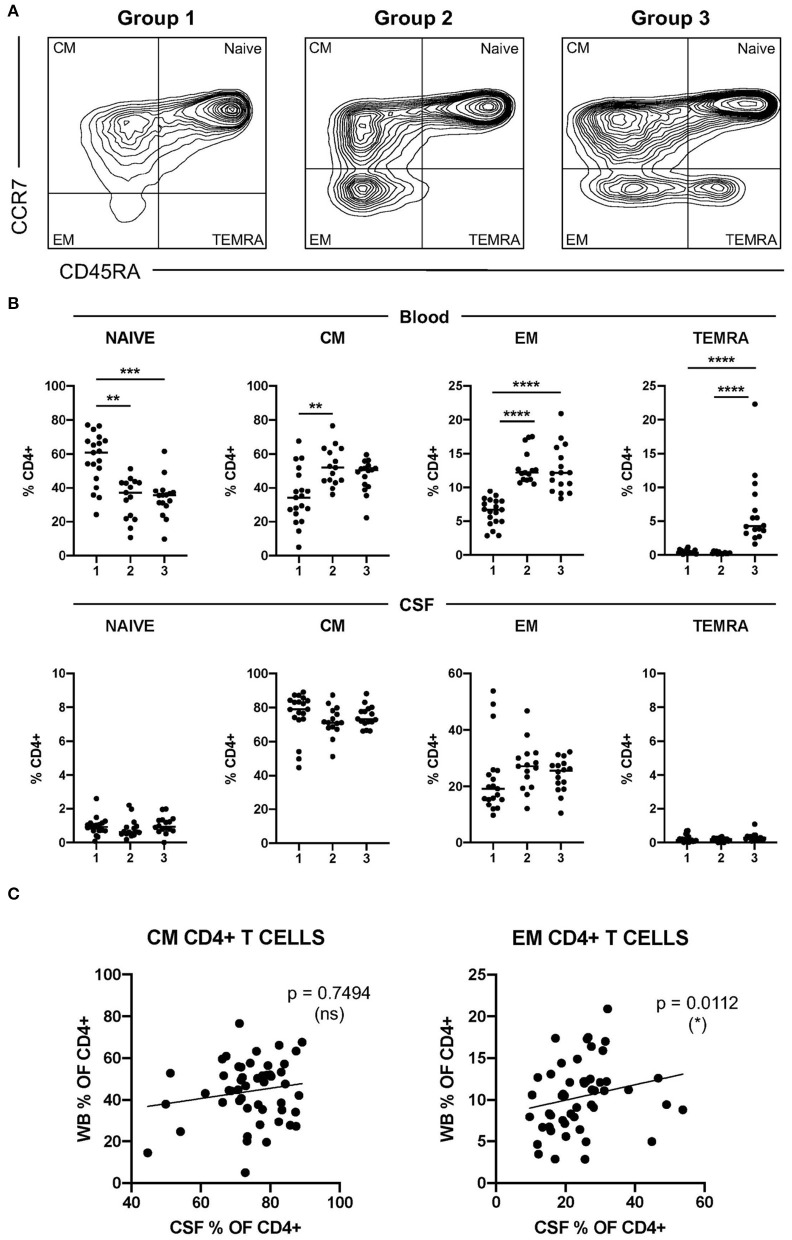
Characterization of CD4+ T-cell senescence using the maturation stage. **(A)** Dot plot showing CCR7 and CD45RA expression on peripheral circulating CD4+ T cells from patients with multiple sclerosis (MS) with low (group 1, left plot), intermediate (group 2, middle plot), and high (group 3, right plot) levels of CD4+ T-cell senescence. **(B)** Frequencies of circulating- and cerebrospinal fluid (CSF)-infiltrating naïve-, CM-, EM-, and terminally differentiated effector memory (TEMRA) CD4+ T cells in patients with MS from groups 1, 2, and 3. **(C)** Correlation between the frequencies in peripheral blood (WB) and CSF of CM- and EM CD4+ T cells from patients with MS. Each dot in the graphs corresponds to a single patient and lines show means. The Kruskal–Wallis test was used to compare patient groups. Linear correlation between variables was tested using Pearson's correlation coefficient. Statistical significance (*p < 0.05, **p < 0.01, ***p < 0.001, and ****p < 0.0001) is shown.

We first identified circulating CD4+ T cells at different maturation stages based on the surface expression of CCR7 and CD45RA [naïve, CCR7+ CR45RA+; central memory (CM), CCR7+ CD45RA-; effector memory (EM), CCR7- CD45RA-; and terminally differentiated effector memory (TEMRA), CCR7- CD45RA+] (Supplementary Figure S1). Patients with MS showed a marked heterogeneity regarding the frequencies of circulating CD4+ T cells at the different maturation stages (Figure 1A). Based on the assumption that TEMRA CD4+ T cells that re-express CD45RA are the most senescent CD4+ T cells and naïve CD4+ T cells the least senescent, we classified patients with MS into three groups representing low (group 1), intermediate (group 2), and high (group 3) level of CD4+ T-cell senescence (Figure 1A). Patients with frequencies of TEMRA CD4+ T cells higher than the mean of all MS patients were classified into group 3. Patients with frequencies of TEMRA CD4+ T cells lower than the mean but with frequencies of EM CD4+ T cells higher than the mean of all patients with MS were classified into group 2. Finally, patients with frequencies of TEMRA- and EM CD4+ T cells lower than the corresponding means were classified into group 1 (Figure 1A). Frequencies of circulating naïve, CM, EM, and TEMRA cells in these patient groups are summarized in Figure 1B. As expected, group 1 contained significantly higher frequencies of naïve CD4+ T cells than groups 2 and 3, but significantly lower frequencies of EM- and TEMRA CD4+ T cells (Figure 1B). We then also analyzed the frequencies of CSF-infiltrating naïve-, CM-, EM- and TEMRA CD4+ T cells in these patient groups (Figure 1B). Naïve- and TEMRA CD4+ T cells were practically absent in all CSF samples (Figure 1B). The frequencies of CSF-infiltrating CM- and EM CD4+ T cells did not show significant differences between groups (Figure 1B). Accordingly, the correlation between the frequencies of circulating and CSF-infiltrating CM- and EM CD4+ T cells was very low or absent (Figure 1C).

### Characterization of CD4+ T-Cell Senescence Based on the Downregulation of CD28 and CD27

**Figure 2:**
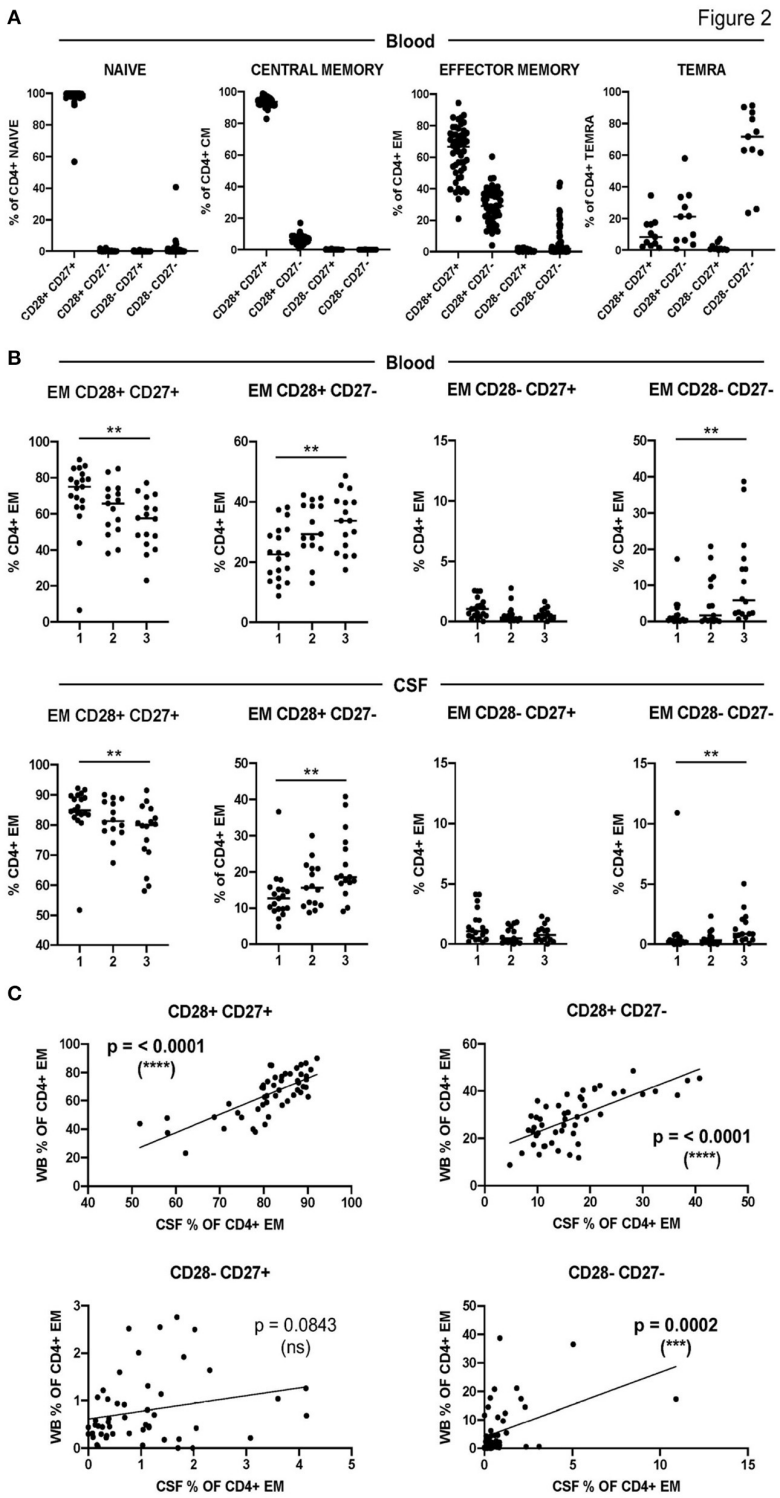
Characterization of CD4+ T-cell senescence using the downregulation of CD28 and CD27. **(A)** Frequencies of CD28+ CD27+, CD28+ CD27-, CD28- CD27+, and CD28- CD27- cells among circulating naïve-, CM-, EM-, and TEMRA CD4+ T cells. **(B)** Frequencies of CD28+ CD27+, CD28+ CD27-, CD28- CD27+, and CD28- CD27- cells among circulating (upper graphs) and CSF-infiltrating (lower graphs) EM CD4+ T cells in the three groups of patients with low (1), intermediate (2), and high (3) levels of CD4+ T-cell senescence. **(C)** Correlation between the frequencies in peripheral blood (WB) and CSF of CD28+ CD27+, CD28+ CD27-, CD28- CD27+, and CD28- CD27- EM CD4+ T cells from patients with MS. Each dot in the graphs corresponds to a single patient and lines show means. The Kruskal–Wallis test was used to compare patient groups. Linear correlation between variables was tested using Pearson's correlation coefficient. Statistical significance (**p < 0.01, ***p < 0.001, and ****p < 0.0001) is shown.

To further characterize CD4+ T-cell senescence in MS, we included the downregulation of CD28 and CD27 molecules in our analysis (Supplementary Figure S1). As expected, the downregulation of CD28 and CD27 costimulatory molecules was associated with the maturation stage of circulating CD4+ T cells (Figure 2A). The terminally differentiated TEMRA CD4+ T cells contained the highest frequencies of CD28- CD27- cells while naïve- and CM CD4+ T cells contained the highest frequencies of CD28+ CD27+ cells (Figure 2A). EM CD4+ T cells showed intermediate frequencies of CD28+ CD27+, CD28+ CD27-, and CD28- CD27- cells, while the frequencies of CD28- CD27+ cells were very low in all the differentiation stages (Figure 2A).

Figure 2B shows the downregulation of CD28 and CD27 in circulating and CSF-infiltrating EM CD4+ T cells from the three groups of patients with different levels of CD4+ T-cell senescence in peripheral blood. As expected, patients from group 1 showed significantly higher frequencies of circulating EM CD28+ CD27+ and significantly lower frequencies of circulating EM CD28+ CD27- and CD28- CD27- than patients from group 3 (Figure 2B). Interestingly, patients from group 1 also showed significantly higher frequencies of CSF-infiltrating EM CD28+ CD27+ and significantly lower frequencies of CSF-infiltrating EM CD28+ CD27- and CD28- CD27- than patients from group 3 (Figure 2B). Accordingly, the frequencies of these cells in peripheral blood and CSF showed strongly significant correlations (Figure 2C). The frequencies of circulating and CSF-infiltrating EM CD28- CD27+ CD4+ T cells did not show any differences between the patient groups or a significant correlation between them (Figures 2B,C).

### Functional Phenotype of CD28+ CD27+ and CD28+ CD27- EM CD4+ T Cells

**Figure 3:**
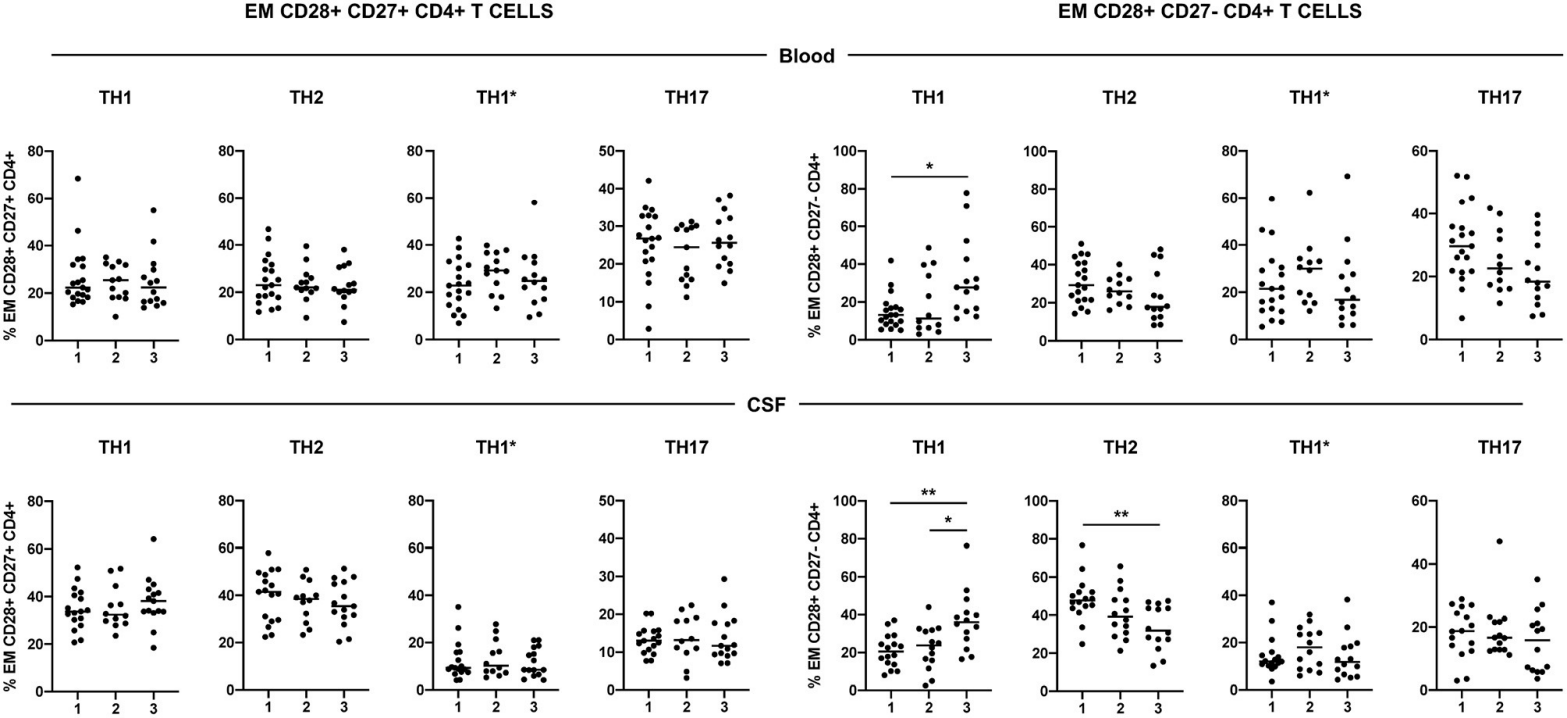
Functional phenotype of CD28+ CD27+ and CD28+ CD27- EM CD4+ T cells. Frequencies of circulating- (upper graphs) and CSF-infiltrating (lower graphs) Th1 (CCR4- CCR6-), Th2 (CCR4+ CCR6-), Th1* (CCR4- CCR6+) and Th17 (CCR4+ CCR6+) EM CD28+ CD27+ (left graphs) and CD28+ CD27- (right graphs) CD4+ T cells. Each dot in the graphs corresponds to a single patient and lines show means. The Kruskal–Wallis test was used to compare patient groups. Statistical significance (*p < 0.05 and **p < 0.01) is shown.

Using the surface expression of chemokine receptors, we classified circulating and CSF-infiltrating CD28+ CD27+ and CD28+ CD27- EM CD4+ T cells into the following functional phenotypes: Th1 (CCR6- CCR4-), Th2 (CCR6- CCR4+), Th1^*^ (CCR6+ CCR4-) and Th17 (CCR6+ CCR4+) (Supplementary Figure S1). The low numbers of CD28- CD27+ and CD28- CD27- EM CD4+ T cells impeded to determine their functional phenotype. The functional phenotype of circulating and CSF-infiltrating CD28+ CD27+ EM CD4+ T cells did not show significant differences between patients with different levels of CD4+ T-cell senescence (Figure 3). However, the frequencies of circulating and particularly of CSF-infiltrating CD28+ CD27- EM CD4+ T cells with a Th1 functional phenotype were significantly higher in patients of group 3. These patients also showed significantly lower frequencies of CSF-infiltrating CD28+ CD27- EM CD4+ T cells with a Th2 functional phenotype (Figure 3).

**Figure 4:**
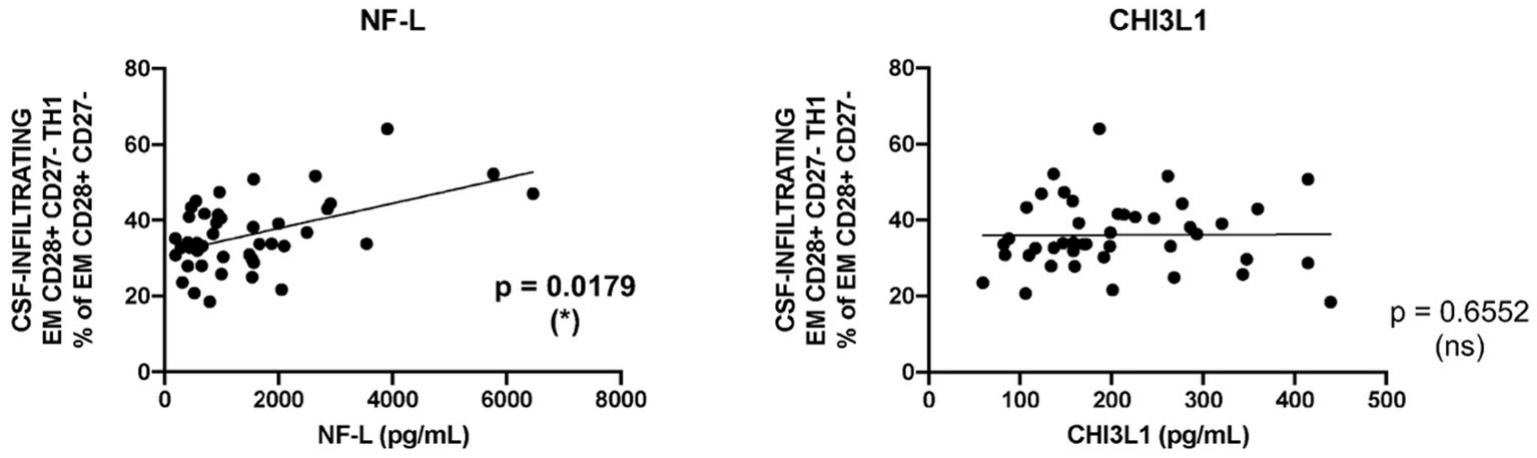
Correlation between CD28+ CD27- EM CD4+ Th1 cells and markers of tissue damage. Correlation between the frequencies of CSF-infiltrating CD28+ CD27- EM CD4+ Th1 cells from patients with MS and the intrathecal amounts of NF-L (left graph) and CHI3L1 (right graph). Each dot in the graphs corresponds to a single patient. Linear correlation between variables was tested using Pearson's correlation coefficient. Statistical significance (*p < 0.05) is shown.

We evaluated CNS damage in our patient cohort with MS using neurofilament light chain (NF-L), a promising intrathecal biomarker of neurodegeneration (28) and chitinase 3-like 1 (CHI3L1), a glycoprotein secreted by activated glia (29). The intrathecal amount of NF-L but not of CHI3L1 showed a significant correlation with the frequencies of CSF-infiltrating EM CD28+ CD27- CD4+ Th1 cells (Figure 4).

### Characterization of Patients With Different Levels of CD4+ T-Cell Senescence

Next, we compared demographical and clinical features of MS patients with different levels of CD4+ T-cell senescence (Table 1). There were no significant differences between patient groups regarding gender, age at the spinal tap, disease duration, clinical course, or the frequency of patients expressing the MS-associated DR15 haplotype. We did not find significant differences neither regarding routine CSF parameters, such as the number of CSF-infiltrating cells, blood-brain barrier (BBB) permeability, or immunoglobulin indices.

**Table 1:** Demographic and clinical features.

		**Level of CD4 senescence**		
	**All**	**1**	**2**	**3**
Number of patients	50	19	15	16
Female/male ratio	1.77	2.16	1.14	1.67
Age (years)	36.2 ± 10.6	34.4 ± 8.2	35.8 ± 9.5	38.7 ± 13.9
Disease duration (days)	1044.8 ± 1869.1	1004.5 ± 1315.9	1786.5 ± 1796.7	1363.0 ± 1906.3
Clinical course				
RIS/CIS # (%)	10 (20)	5 (26.3)	4 (33.3)	1 (6.2)
RRMS # (%) in review	36 (72)	14 (73.7)	8 (53.4)	14 (87.6)
PMS^*^ # (%)	4 (8)	0	3 (13.3)	1 (6.2)
DR15 (% patients)	22 (44)	11 (57.8)	6 (40)	5 (31.2)
CSF				
CSF cell count (cells/uL)	7.08 ± 7.7	6.11 ± 3.9	9 ± 12.2	6.53 ± 6.16
BBB damage^**^ (% patients)	11 (22)	4 (21)	2 (13.3)	5 (31.2)
IgG index	1.02 ± 0.7	1.06 ± 0.5	1.12 ± 1.02	0.87 ± 0.48
IgM index	0.15 ± 0.19	0.12 ± 0.13	0.20 ± 0.28	0.16 ± 0.15
IgA index	0.34 ± 0.28	0.41 ± 0.45	0.28 ± 0.04	0.31 ± 0.04

* *PMS, secondary progressive MS and primary progressive MS*.

** *Blood–brain barrier (BBB) damage (QALB-QNORM > 0)*.

### Comparison of CD4+ T-Cell Senescence in Patients With MS and Controls

**Figure 5:**
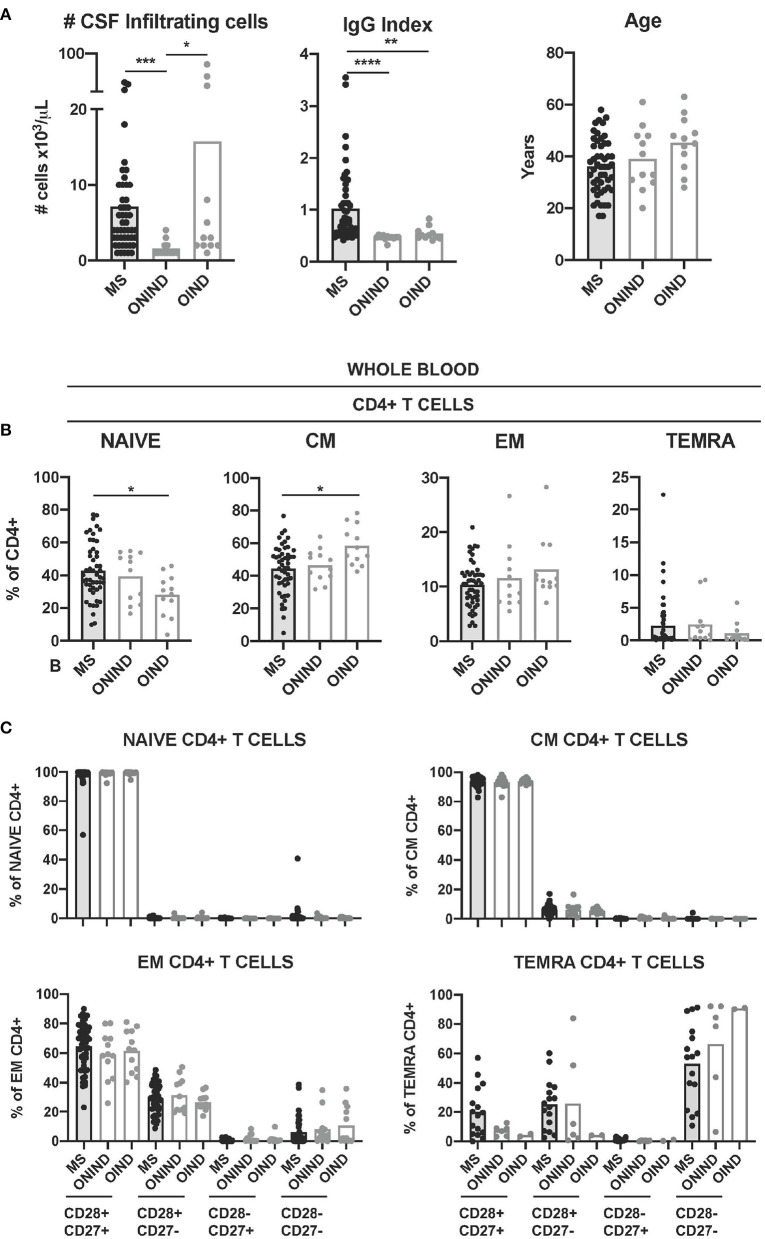
Comparison of circulating CD4+ T-cell senescence between patients with MS and controls. **(A)** Graphs showing the number of CSF-infiltrating cells (left graph), IgG Index (middle graph) and age in years (right graph) in patients with MS (*n* = 50) and control patients affected of other non-inflammatory neurological disease (ONIND) (*n* = 12) and other inflammatory neurological disease (OIND) (*n* = 12). **(B)** Frequencies of circulating naïve-, CM-, EM-, and TEMRA CD4+ T cells in patients with MS and controls. **(C)** Frequencies of CD28+ CD27+, CD28+ CD27-, CD28- CD27+, and CD28- CD27- cells among circulating naïve-, CM-, EM-, and TEMRA CD4+ T cells from patients with MS and controls. Each dot in the graphs corresponds to a single patient and bars show means. The Kruskal–Wallis test was used to compare patients with MS and controls. Statistical significance (*p < 0.05, **p < 0.01, ***p < 0.001, and ****p < 0.0001) is shown.

Finally, we compared CD4+ T-cell senescence in patients with MS and controlled affected by ONINDs and OINDs from whom CSF and paired blood samples were available. Patients with MS and patients with OIND showed significantly higher numbers of CSF-infiltrating T cells than patients with ONIND, while only patients with MS showed a significantly higher immunoglobulin G (IgG) index (Figure 5A). Although OIND was older, age differences did not reach statistical significance.

The frequencies of circulating naïve CD4+ T cells in patients with MS were significantly higher than in patients with OIND, while the frequencies of CM CD4+ T cells were lower (Figure 5B). The younger age of patients with MS might be the reason for these differences, since the frequency of both the cell subtypes in blood correlated with age (Supplementary Figure S2). We did not find significant differences between patients with MS and controls for circulating EM- and neither for TEMRA CD4+ T cells (Figure 5B). The frequencies of circulating CD28+ CD27+, CD28+ CD27-, CD28- CD27+, and CD28- CD27- CD4+ T cells at the different maturation stages (naïve, CM, EM, and TEMRA) from patients with MS and controls did not show statistically significant differences either (Figure 1C).

**Figure 6:**
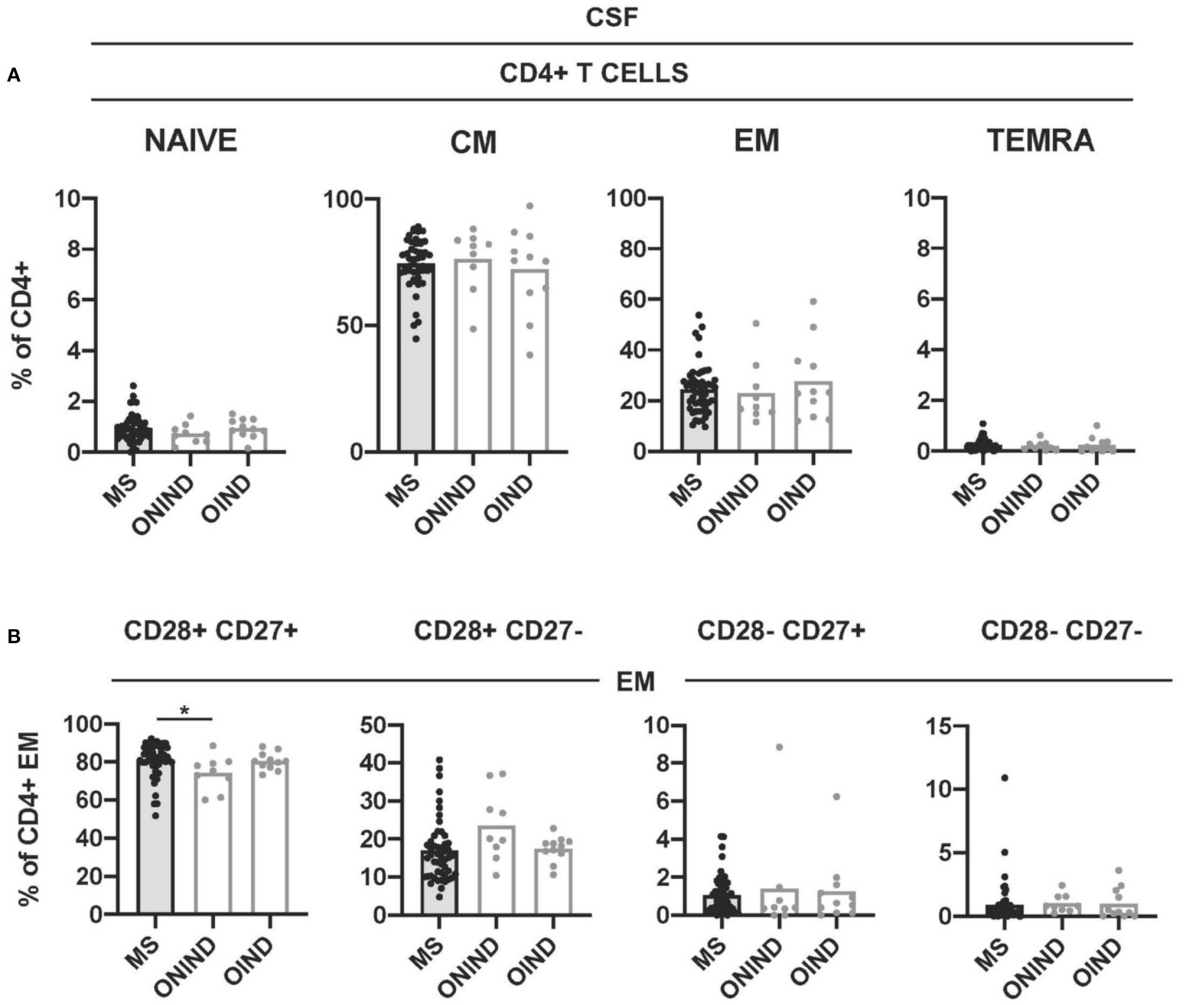
Comparison of intrathecal CD4+ T-cell senescence between patients with MS and controls. **(A)** Frequencies of CSF-infiltrating naïve-, CM-, EM-, and TEMRA CD4+ T cells in patients with MS and controls. **(B)** Frequencies of CD28+ CD27+, CD28+ CD27-, CD28- CD27+, and CD28- CD27- cells among CSF-infiltrating EM CD4+ T cells from patients with MS and controls. Each dot in the graphs corresponds to a single patient and bars show means. The Kruskal–Wallis test was used to compare patients with MS and controls. Statistical significance (*p < 0.05) is shown.

We further compared CD4+ T cell senescence in freshly isolated CSF-infiltrating CD4+ T cells from patients with MS and controls. Naïve- and TEMRA CD4+ T cells were practically absent in all CSF samples (Figure 6A). The frequencies of CM- and EM CD4+ T cells did not show significant differences between patients with MS and controls (Figure 6A). We then compared the frequencies of CSF-infiltrating CD28+ CD27+, CD28+ CD27-, CD28- CD27+, and CD28- CD27- EM CD4+ T cells in patients with MS and controls. Only the frequencies of CD28+ CD27+ EM CD4+ T cells were significantly higher in patients with MS compared with ONIND (Figure 6B).

## Discussion

Antigen-induced T-cell exhaustion and T-cell senescence are considered as important regulatory mechanisms controlling immune responses mediated by effector T cells (1). In malignant tumors, these mechanisms are enhanced and allow the tumor to evade the immune system (4), while they might be reduced in autoimmunity. MS is considered an autoimmune disease of the CNS, in which immune responses mediated by autoreactive CD4+ T cells seem to play a crucial role (13). Inadequate T-cell exhaustion (12, 16) and maybe also T-cell senescence might facilitate these autoreactive responses in patients with MS. With the aim to better understand antigen-induced T-cell senescence in MS and its putative role in disease pathogenesis, we have characterized CD4+ T-cell senescence in patients with MS and controls.

In a first step, we used the surface expression of CCR7 and CD45RA to analyze the maturation stage of circulating CD4+ T cells in patients with MS. At this first level of analysis, we found a high degree of heterogeneity in frequencies of naïve-, CM-, EM-, and TEMRA CD4+ T cells between patients with MS and, therefore, grouped patients into low (group 1), intermediate (group 2), and high (group 3) level of CD4+ T-cell senescence. The analysis of CSF-infiltrating cells demonstrated that mainly CM- and EM CD4+ T cells cross the BBB and that their frequencies in the CSF were not associated with their frequencies in peripheral blood suggesting a selective recruitment of CM- and EM CD4+ T cells into the CNS. By combining the maturation stage and the downregulation of CD28 and CD27 costimulatory molecules, we further defined 16 putative stages of CD4+ T cell senescence in which naïve CD28+ CD27+ cells should be the least and TEMRA CD28- CD27- cells the most senescent T cells. Supporting that this second analysis to evaluate CD4+ T-cell senescence is sound, we found that CD28- CD27- cells were more abundant among circulating TEMRA CD4+ T cells and CD28+ CD27+ among circulating naïve- and CM CD4+ T cells. Circulating EM CD4+ T cells, with an intermediate maturation stage correspondingly showed intermediate levels of CD28+ CD27+ and CD28- CD27- cells. T-cell senescence is considered a regulatory mechanism of effector cells, and accordingly, the downregulation of CD28 and CD27 molecules was found mainly for EM and TEMRA cells. This second analysis confirmed the heterogeneity of the patients and supported our initial classification into the three groups. Patients from group 1 showed significantly higher frequencies of circulating EM CD28+ CD27+ and lower frequencies of CD28+ CD27- and CD28- CD27- cells than patients from group 3. Interestingly, the frequencies of circulating- and CSF-infiltrating CD28+ CD27+, CD28+ CD27-, and CD28- CD27- EM CD4+ T cells correlated strongly. These results suggest that, while the CD4+ T-cell maturation stage influences migration through the BBB, the downregulation of CD28 and CD27 does not seem to influence this migration.

Circulating- and CSF-infiltrating CD28- CD27+ CD4+ T cells showed comparably low frequencies for all maturation stages. Furthermore, while the frequencies of circulating and CSF-infiltrating CD28+ CD27+, CD28+ CD27-, and CD28- CD27- EM CD4+ T cells were significantly different between groups 1 and 3, the frequencies of circulating- and CSF-infiltrating CD28- CD27+ EM CD4+ T cells were comparable in the three groups of patients. These results suggest that CD28- CD27+ cells most likely do not represent a senescent stage and that the induction of senescence in CD4+ T cells probably always starts with the downregulation of CD27 and only then of CD28.

As mentioned earlier, the induction of T-cell senescence is considered as a regulatory mechanism to control immune responses. We can assume that cells downregulating CD28 and CD27 molecules are cells that underwent more rounds of antigen stimulation *in vivo* and, therefore, are cells that are likely relevant in disease pathogenesis. In this context, the use of markers of T-cell senescence might facilitate the identification of relevant pathogenic T cells and the detailed characterization of these pathogenic T cells including the determination of their specificity is crucial to better understand MS and to develop new therapeutic approaches. Supporting the assumption that markers of T-cell senescence might be useful to identify pathogenic T cells in MS, it is important to note that CD28+ CD27- EM CD4+ T cells with a proinflammatory Th1 functional phenotype that were significantly more frequent in patients of group 3 showed a correlation with the intrathecal amount of NF-L, a biomarker of CNS damage. Interestingly, CD28+ CD27- TEMRA and EM CD4+ T cells expressing Th1- and cytotoxicity-associated genes have also been described to be increased and associated with higher damage in rheumatoid arthritis patients (17, 30). Furthermore, we found that MS patients with intrathecal CD4+ T-cell reactivity against GDP-L-fucose synthase derived peptides and that are characterized by higher neuroinflammation and neurodegeneration, also showed higher frequencies of CD28+ CD27- TEMRA and EM CD4+ T cells expressing Th1- and cytotoxicity-associated genes (31). Altogether these data support a pathogenic role of CD28+ CD27- TEMRA and EM CD4+ T cells in autoimmunity. Furthermore, data supporting a role of Th1 cells in MS are: (i) an increased autoproliferation of CM- and EM CD4+ Th1 cells (32), (ii) higher frequency of myelin basic protein (MBP)-specific Th1+ CD4+ T cells in patients with MS (33), (iii) higher sensitivity of naive CD4+ CD45RA+ to activation by MBP (34), and (iv) involvement of a Th1 axis between T cells and CD11c+ B cells in MS (35).

Due to the dual meaning of markers of T-cell senescence on CD4+ T cells, i.e., regulation vs. the previous activation, it is difficult to discern whether a high level of CD4+ T-cell senescence in some patients with MS reflects better T-cell regulation in these patients or a higher antigen-specific T-cell activation. The fact that patients from group 3 showed higher frequencies of CSF-infiltrating CD28+ CD27- EM CD4+ Th1 cells associated with neurodegeneration, suggests that a high level of CD4+ T-cell senescence most likely reflects higher antigen-specific T-cell activation. In this study, however, we have not been able to associate a high level of CD4+ T-cell senescence with high-disease activity to support this hypothesis. The limited clinical data regarding disability evolution and imaging findings that were available rendered an analysis to associate markers of senescence with the level of disease activity impossible. We think that further research in a large cohort of well-characterized patients is required to determine whether markers of T-cell senescence reflect higher T activation or T-cell regulation and what they mean for understanding T-cell senescence/activation in MS.

Our comparison of patients with MS and controls did not demonstrate a defective or accelerated T-cell senescence in patients with MS compared with controls. Further research in a larger number of control samples should be pursued to clarify putative defects in T-cell senescence in MS.

In summary, our results suggest that T-cell senescence most likely contributes to controlling autoimmune responsiveness in MS. An in-depth characterization of CD4+ T-cell senescence in patients with MS by combining the maturation stage and the downregulation of CD28 and CD27 costimulatory molecules might therefore facilitate a more detailed characterization of pathogenic CD4+ T cells. Further research should be pursued to determine the validity of these markers to identify patients with more aggressive forms of the disease and also to clarify whether T-cell senescence is compromised or not in patients with MS.

## Data Availability Statement

The original contributions presented in the study are included in the article/Supplementary Material, further inquiries can be directed to the corresponding author/s.

## Ethics Statement

The studies involving human participants were reviewed and approved by Cantonal Ethics Committee of Zurich (EC-No. 2013-0001). The patients/participants provided their written informed consent to participate in this study.

## Author Contributions

PT-O and MP: major role in the acquisition of data. CC and MD: acquisition of data. RM: interpreted the data and revised the manuscript for intellectual content. MS: design and conceptualized study, analyzed the data, interpreted the data, and drafted the manuscript for intellectual content. All authors contributed to the article and approved the submitted version.

## Funding

Funding was taken care of by the European Research Council Advanced Grant (340733) (RM), Clinical Research Priority Programs (CRPPs) Heterogeneity MS and Precision MS of the University Zurich (RM and MS), Clinical, Swiss National Science Foundation (Sinergia UnmetMS grant number: CRSII3_154483) (RM and MS), and the Swiss MS Society (RM).

## Conflict of Interest

The authors declare that the research was conducted in the absence of any commercial or financial relationships that could be construed as a potential conflict of interest.

## Publisher's Note

All claims expressed in this article are solely those of the authors and do not necessarily represent those of their affiliated organizations, or those of the publisher, the editors and the reviewers. Any product that may be evaluated in this article, or claim that may be made by its manufacturer, is not guaranteed or endorsed by the publisher.
