# Supplementary material for: Characterization of Antigen-Induced CD4+ T-Cell Senescence in Multiple Sclerosis
Source: Front Neurol. 2022 Feb 3;13:790884. doi: 10.3389/fneur.2022.790884 (PMC8852676; doi:10.3389/fneur.2022.790884)
Supplement: Supplementary file 1 [file Data_Sheet_1.PDF]

Table S1. Demographic and clinical features of patients and controls.

	PATIENTS					
	MS				ONIND***	OIND****
	All	RIS/CIS	RRMS	PMS**	All	All
**# of Patients**	50	10	36	4	12	12
**Female/Male Ratio**	1.77	1.5	1,76	3.0	1.4	0.5
**Age (y)***	36.2 ± 10.6	36.6 ± 10.5	31.6 ± 8.8	49.5 ± 4.6	39.1 ± 11.9	45.2 ± 10.2

* Mean ± standard deviation is shown.
**PMS, progressive MS including SPMS and PPMS.
***ONIND (5, migraine; 3, psudotumor cerebri; 2, amyotrhophic lateral sclerosis; 1, Parkinson; 1 multi system atrophy).
****OIND (2, borreliosis; 1, Sjögren's syndrome; 3, meningoencephalitis; 2, Chronic inflammatory demyelinating polyneuropathy; 2, Rhombencephalitis; 1, Tolosa-Hunt Syndrome sarcoidosis; 1, Lymbic Encephalitis)

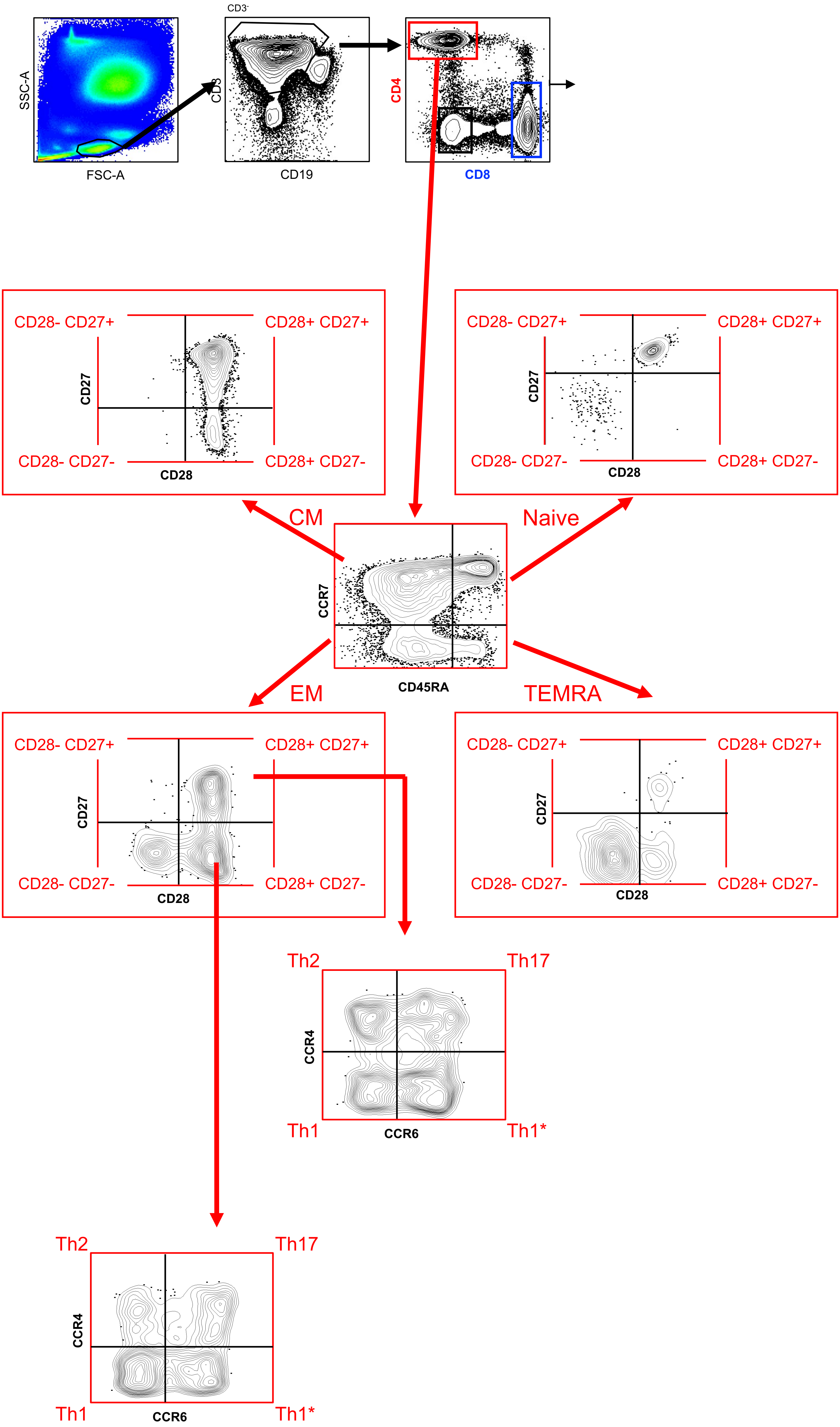
SSC-A
FSC-A
CD3⁻
CD3
CD19
CD4
CD8
CD28- CD27+
CD28+ CD27+
CD27
CD28- CD27-
CD28+ CD27-
CD28
CM
Naive
CCR7
CD45RA
EM
TEMRA
Th2
Th17
CCR4
Th1
CCR6
Th1*

Figure S1. Gating strategy. First doublets are excluded, followed by identification of lymphocytes by size. Next, CD3+ T cells are identified and among them, CD3+ CD4+ cells. Then CD4+ T cells are separated in CM (CCR7+ CD45RA-), EM (CCR7- CD45RA-), TEMRA (CCR7- CD45RA+) and naive (CCR7+ CD45RA+). Naive, CM, EM and TEMRA CD4+ T cells are then separated in CD28+ CD27+, CD28+ CD27-, CD28- CD27+ and CD28- CD27-. Finally, CD28+ CD27+ and CD28+ CD27- EM CD4+ T cells are separated in Th1 (CCR6- CCR4-), Th2 (CCR6- CCR4+), Th1* (CCR6+ CCR4-) and Th17 (CCR6- CCR4+) cells. <u>Antibodies</u>: anti-CD3 AF700, anti-CD4 PE TR, anti-CD8 BV510, anti-CD45RA BV711, anti-CCR7 BV421, anti-CD27 APC Cy7, anti-CD28 PE Cy7, anti-CCR4 APCand anti-CCR6 BV785.

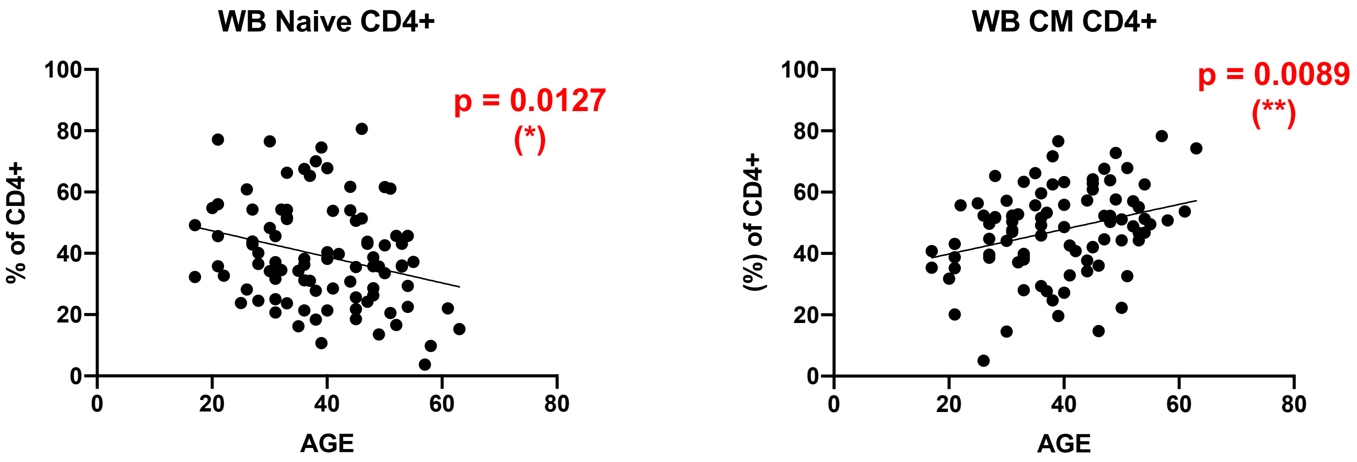

Figure S2. Correlation between age and frequencies of circulating Naive and CM CD4+ T cells. Correlation between the frequencies of circulating naive (left graph) and CM (right graph) CD4+ T cells and age. Both graphs represent the 50 MS patients, 12 ONIND and 12 OIND controls. Each dot in the graph correspond to a single patient. Linear correlation between variables was tested using Pearson's correlation. Statistical significance (* p<0.05 and ** p<0.01) is shown.
